# The Impact of Modifications in the Testing Method for Determining Lean Meat Content in Carcasses on Pig Slaughter Value in Poland

**DOI:** 10.3390/ani15071016

**Published:** 2025-04-01

**Authors:** Dariusz Lisiak, Piotr Janiszewski, Karol Borzuta, Eugenia Grześkowiak, Krzysztof Powałowski, Łukasz Samardakiewicz, Beata Lisiak

**Affiliations:** Department of Meat and Fat Technology, Prof. Wacław Dąbrowski Institute of Agricultural and Food Biotechnology—State Research Institute, 60-111 Poznań, Poland; dariusz.lisiak@ibprs.pl (D.L.); karol.borzuta@ibprs.pl (K.B.); eugenia.grzeskowiak@ibprs.pl (E.G.); krzysztof.powalowski@ibprs.pl (K.P.); lukasz.samardakiewicz@ibprs.pl (Ł.S.); beata.lisiak@ibprs.pl (B.L.)

**Keywords:** pork carcasses, meatiness, verification equations, CGM device

## Abstract

In order to harmonize carcass grading, it was necessary to make sure that all methods used in the EU for assessing lean meat content give the same result when dissecting the same carcass. Research was conducted to establish the influence of the changes to the testing method introduced by the EU in 2018 on the meatiness value of pig carcasses. A question arose about whether this system would affect the results and, thus, change the lean meat content of domestic fattening pigs compared to the regression equations that are currently valid. In this study, the CGM-Sydel device was used. It was found that the form of the regression equation was not significantly affected by the new test procedure because the obtained SF (scaling factor), being equal to 0.89, was the same as the one used earlier by EU regulations. The change in the form of the regression equation was affected by the increase in the meatiness level of pig carcasses and their weight in the period from 2011 to 2023 in Poland. The implementation of the new equation for the CGM-device caused of the level of meatiness to be 0.58 pp.

## Simple Summary

In order to harmonize carcass grading in the SEUROP system, it was necessary to make sure that all of the methods used in assessing lean meat content give possibly the same results when dissecting the same carcass. It was important that this evaluation affected the results of dissection and, thus, changed the lean meat content of domestic fattening pigs compared to the regression equations that are currently in force. The aim of this study was to investigate the impact of the current modification of the testing procedure on the level of lean meat content in domestic fattening pigs compared to the procedure that was in force in 2018. The research was carried out on an opto-needle device that is often used in the Polish meat industry. After the completion of this study, it was concluded that the new procedure for testing grading devices introduced by the EU in 2018 did not affect the form of the regression equation for estimating lean meat content in Poland.

## 1. Introduction

The pork carcass grading system has been in use in the European Union since 1984. This system is known as SEUROP, in which the pork carcass quality grade is determined by the hot carcass weight and the lean meat contents that have been cut with a knife by different deboning butchers, presented as a percentage of the hot carcass weight [1]. In practice, this system is based on the objective measurement of various anatomical points of the carcass, taken by using various devices and highly correlated with the meat content. The lean meat content assessment is carried out using statistics (regression equations) developed for each device on the basis of the dissection of strictly selected carcasses. In accordance with Implementing Decision (EU) No. 2024/1434 approving techniques for grading pig carcasses in Poland and by Repealing Decision 2005/240/EC, the following manual devices are permitted for use in Poland: optical-needles—CGM (Sydel, Paris, France) IM-03 (BSA, Poznań, Poland), Fat-O-Meater II (FOM II, Frontmatec, Kolding, Denmark); ultrasonic—Ultra Fom 300, (Frontmatec, Kolding, Denmark); manual method (ZP, Poznań, Poland) and automatic, ultrasonic—Autofom III and IV (Frontmatec, Kolding, Denmark); automatic, vision—CSB Image Meater 2.0 (CSB-System International, Inc., Woodstock, GA, USA) EstiMeat Expert” and EstiMeat Pro devices (EstiTech Sp. z o.o. Kłoda Duża, Poland).

Since the beginning of classification device testing, the procedure of full dissection of the left half carcass, adapted from a German method called total dissection, has been in use, causing a significant increase in the workload of this method [2,3]. Therefore, in 1994, a simplified method of dissection was introduced, considered as a reference method, in which four basic carcass cuts and a tenderloin are dissected and the meat content in the carcass is determined based on the equation in [4], with the result initially corrected by the correction factor SF = 1.3 and then starting from 2008 by SF = 0.89 [3]. Currently, as a result of research on the use of X-ray scanners and computed tomography methods to assess the meat content in carcasses, it was determined that this method is very accurate (0.51% lean meat content) and can be also used as a reference method [2,3,5]. In 2018, this method was recognized as the third way to determine the lean meat content of pork carcasses for the purpose of testing classification devices.

In order to harmonize carcass grading, it is necessary to make sure that all of the methods for assessing lean meat content give possibly the same results when dissecting the same carcass. As established by Daumas [6], all efforts put into harmonising pork carcass grading in EU countries for the past 20 years have not brought sufficient solutions. For 20 different classification devices, which are used in EU countries, 126 different regression equations with an estimation error (RMSEP) of 1.1 to 2.5% lean meat content (LMP) were developed. But, at the time of that study, only eight countries had adapted their classification devices based on the last changes in the testing procedure from 2018. These changes primarily involved the obligation to calculate the SF correction factor for the results of simplified dissection or the results of computed tomography assessment at each test of the grading device. A question then arises regarding whether this system will affect the dissection results and, thus, will it change the lean meat content of domestic fattening pigs compared to the regression equations that are currently valid? The aim of this study was to investigate the impact of the current modification of the testing procedure, taking into account the development of the reference coefficient (SF), on the level of lean meat content in domestic fattening pigs compared to the procedure that was used in 2018. The research was carried out with the CGM (Sydel, France) optical-needle device that is often used in the Polish meat industry. The importance of the research is underlined by the fact that over 90% of pig carcasses slaughtered in Poland have been classified in the SEUROP system.

## 2. Materials and Methods

The research was carried out in a few stages, which are described below. Generally, the CGM-Sydel classification device was tested according to the changed procedure from 2018, and then the results of the assessment of lean meat content in domestic carcasses were estimated according to the regression equations from 2011 [7] and the developed equation from 2023 [8] and compared.

### 2.1. Method of Selecting Carcasses for This Study

The biological variability in pigs slaughtered in Poland was analyzed in August and November 2022 and January 2023 based on 882,475 carcasses. Because in Poland, so far, the share of slaughtering entirely males amounts to a very small percentage of pigs, it was decided that the selection of the sample be based on sex, with 50% gilts and 50% castrated pigs. The carcass measurements were taken in the following 7 polish slaughterhouses representing the average national population of fatteners: Animex Foods branches in Ełk, Szczecin (Poland), and Starachowice (Poland); the meat processing plant Zakłady Mięsne “Dobrosławów” in Puławy (Poland), the slaughterhouse “Przywarty” in Strzałkowo (Poland), the slaughterhouse “Rytel” in Łomża (Poland), and the meat processing plant “Tur” in Łapanów (Poland).

Backfat thickness in point F (the thickness of backfat with skin in millimetres measured at the third and fourth last rib position, 6 centimetres from the dorsal midline, measured parallel to the split line of the carcass) was measured with the CGM-Sydel optical-needle device. The left half carcasses were measured by a qualified carcass grader at the end of the slaughter line no later than 45 min after the pigs had been stunned. The measurement results are presented below in Table 1.

Based on the mean results for backfat thickness and hot carcass weight and the standard deviation, the population of carcasses was divided into 3 groups according to the values presented in Table 2 and Table 3.

The numbers of required and used carcasses for dissection are shown below in Table 4. The selection of the carcasses and dissection were carried out at the Meat Plant “Skiba” in Chojnice (Poland). On the slaughter line, 126 carcasses with hot carcass weights varying from 60 to 120 kg were selected according to the groups described in Table 2, Table 3 and Table 4. Carcasses were selected taking into consideration two factors: backfat thickness at point F measured using an optical-needle CGM device (Groups 1–3) and hot carcass weight (Groups A–C). Additionally, proper carcass splitting into half carcasses, as well as the lack of damage on the carcasses (veterinary confiscation), was taken into account. The carcasses were selected on the slaughter line no later than 45 min after the pigs had been stunned.

As shown in Table 4, the required (values in brackets) and performed numbers of half carcasses were agreed for partial (126) and total (12) dissection. Full conformity was also obtained in all of the research subsamples for total dissection. However, in the case of partial dissection, there were a few differences, ranging from 1 to a maximum of 3 in the half carcasses, as well as in some subsamples, between the required and performed numbers of the samples. But this did not cause any significant differences in the required number ratio even in the three groups with different fat thicknesses and in the three groups with different weights (ratio of 1:2:1). So, the required and performed numbers of the half carcasses in the subgroups may have been as small, without having an influence on the regression equations’ form.

### 2.2. Measurements and Dissection

All of the selected carcasses were manually measured by an operator with a CGM device. The selected carcasses were marked and the thicknesses of the backfat and longissimus dorsi muscle were measured using the tested device method described by Lisiak et al. [9].

A team of four experienced butchers carried out the dissections. Partial dissection of the left side was carried out on all of the 126 carcasses using the EU reference method [4] 24 h after slaughter. Four main cuts were made into muscles, fat, and bones. Lean meat content was calculated according to the following equation for partial dissection [10]:

YPD = 100 × (weight of tenderloin + weight of lean meat in the shoulder, loin, ham, and belly)/weight of tenderloin + weight of shoulder, loin, ham, and belly before dissection.

### 2.3. New Procedure Compared to the Previous Regulation

The reference lean meat content was finally calculated taking into account the scaling factor SF (bias correction), which was calculated based on the total dissection of the subsample of the carcasses (SF = YTD/YPD):

LMP_ref_ = YPD × SF

For the calculation of the scaling factor (SF), total dissection was carried out on the subsample of the carcasses (*n* = 12) and the lean meat content was calculated according to the following equation for total dissection [10]:

YTD = (100 × weight of lean meat)/half carcass weight

### 2.4. Statistics

The regression equations for the tested devices were developed using the PLS-PCR (Partial Least Squares Principal Components Regression) procedure, which is permitted for use by EU regulations [11]. The assumption of this method is that the prediction error is minimized by using functions and predictors, which explain the variability in the sample and the linear model of regression in the most comprehensive way. Estimation accuracy was rated using the RMSEP (root mean square error of prediction) indicator calculated with the PRESS statistic, which complies with cross-validation (SAS v. 9.2) An RMSEP lower than 2.5 was proposed for the authorization of every tested device. The developed regression equation for the CGM device was used to evaluate the LMP content of the domestic pig population from the 7 slaughterhouses described above, and the results were compared with the older regression equation from 2011 [9], which has following form: LMP _CGM_ = 59.42 + 0.1322M2 − 0.6275F2.

## 3. Results

### 3.1. Results of Carcass Weight and Fat Thickness Measured by a CGM Device and Lean Meat Percentage Obtained by Dissection

A descriptive summary of the characteristics of the selected carcasses for testing using a CGM device is given below in Table 5, Table 6, Table 7 and Table 8.

For total samples, the measured features were as follows:

- Hot carcass weight—97 kg (*n* = 126; SD = 12.26; min. 70.4 kg; max. 120 kg);
- fat thickness by CGM—15.1 mm (*n* = 126; SD = 5.07; min. 7.0 mm; max. 31.0 mm);
- reference LMP 58.8% (*n* = 126; SD = 4.01; min. 48.7%; max. 66.8%).

The animals were originally from 49 farms. A maximum of FOUR animals came from one farm. The average number of animals per farm was 2.6. The small number of fatteners selected for the dissection test from individual farms was possibly a result of the low productivity of these farms (about a hundred pigs per year). In the farms, there were various pig breeds, e.g., Polish Large White, Polish Landrace, Duroc, Hampshire, Pietrain, Line 990, and their hybrids, as well as foreign hybrid lines, used in production. For these reasons, it was proved that the dissection material was highly bioheterogenous and representative of the native pig population.

The average lean meat percentages of total and partial dissection and the scaling factor were as follows:

YTD = 59.62;
YPD = 67.12;
SF = 0.89.

**Figure 1:**
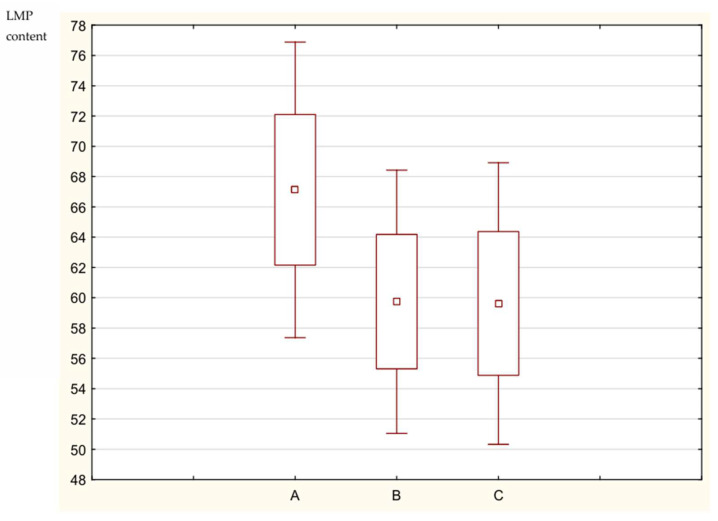
LMP content dependent on the dissection method. Explanation: A—LMP acc. Partial dissection without SF. B—LMP acc. Partial dissection with SF. C—LMP acc. Total dissection.

In Table 9, we can see the individual results of LMP obtained by the dissection of subsample carcasses (*n* = 12) stated for total and partial dissection without and with the scaling factor. It must be stated that the difference between LMP content in the case of total and partial dissection is very high, differing by 7.5 pp, but after taking into account SF, the meatiness from partial dissection is almost the same as that from total dissection (difference of 0.12 pp), as shown below in Figure 1.

**Figure 2:**
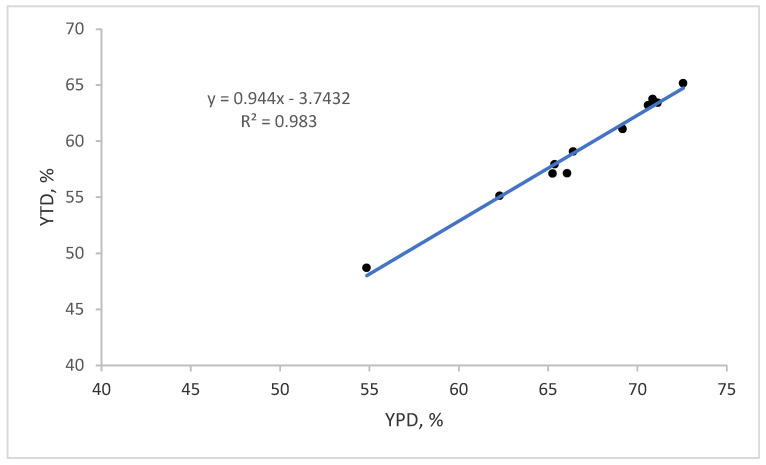
Total dissection (YTD) % vs. partial dissection (YPD) % (*n* = 12).

The correlation between the results of the two types of dissection is very high, as we can see in Figure 2. But in the case of partial dissection, meatiness without SF was higher by 7.50 pp, which confirms the application of reasonableness in the scaling factor.

### 3.2. Elaboration of the Regression Equation for the CGM Device

As a result of the test for the CGM device, the following regression equation is used:

Y_LMP_ = 60.7538 − 0.6465F2 + 0.1243M2

where the following definitions are used:

F2—Fat depth measured at the third to the fourth rib from the last rib position, 6 cm from the dorsal midline, measured parallel to the split line of the carcass;

M2—Loin depth measured at the third to fourth rib from the last rib position, 6 cm from the dorsal midline, measured parallel to the split line of the carcass.

#### RMSEP_(*n*=126)_ = 2.22

**Figure 3:**
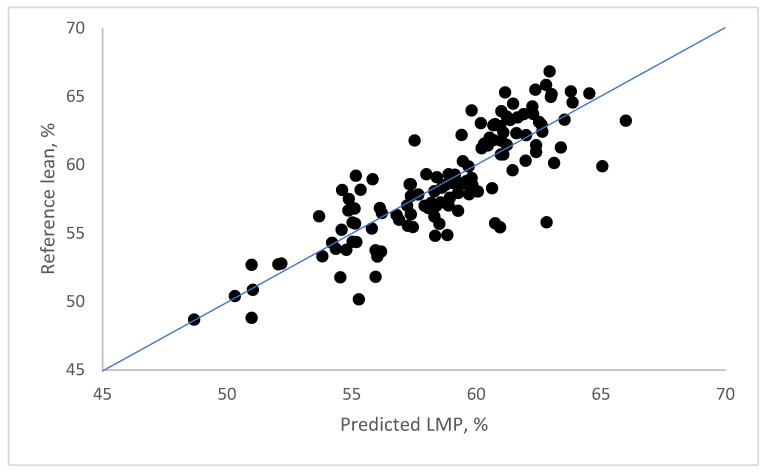
Reference vs. CGM predicted lean meat content, % (*n* = 126).

As illustrated in Figure 3, the correlation between LMP content determined by reference dissection and predicted by the above regression equation is very high. The obtained limit of RMSEP is below 2.5.

### 3.3. A Comparison of the Results of the Lean Meat Content Assessment of Domestic Fattening Pigs According to the Old and New Regression Equations

The material collected from the assessment of the pork carcasses performed by seven slaughterhouses included measurements of the traits taken into account when testing the CGM device, i.e., backfat thickness at point F1 and the longissimus dorsi muscle thickness at point M2. This allows for calculating the lean meat content in carcasses based on the regression equations from 2011 and 2023. The studied population of 882,475 pork carcasses was characterized by the following structure of meat classes determined using the old regression equation:

S class 47.22% (429,534 carcasses);
E class 43.87% (399,115 carcasses);
U class 5.34% (48,359 carcasses);
R class 0.53% (4841 carcasses);
O class 0.04% (350 carcasses);
P class 0.01% (96 carcasses).

**Figure 4:**
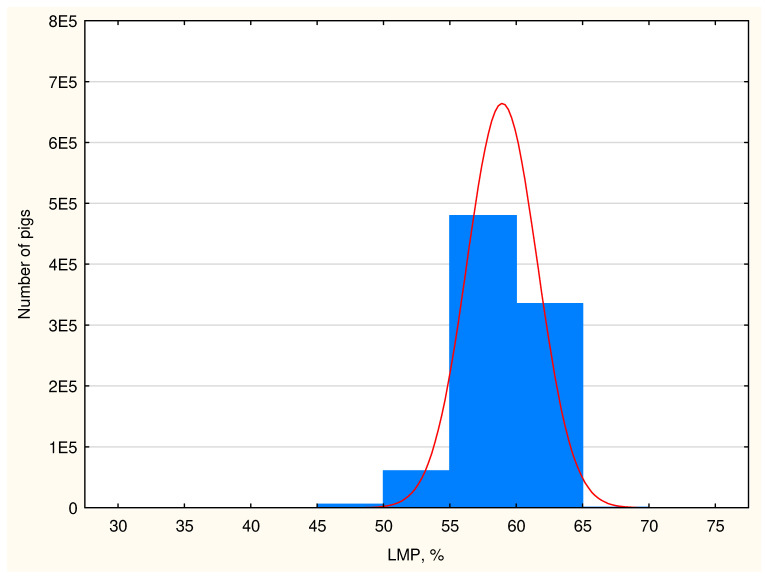
The meatiness structure (blue columns) and distribution curve (red) of the Polish pig population according to the regression equation from 2011 (1E5 means 1 × 10^5^ etc.).

**Figure 5:**
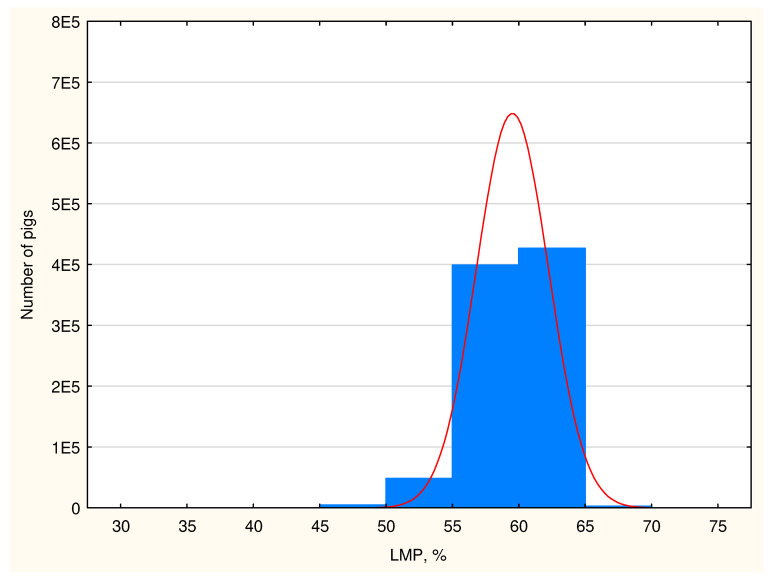
The meatiness structure (blue columns) and distribution curve (red) of the Polish pig population according to the regression equation from 2023 (1E5 means 1 × 10^5^ etc.).

The average results of the measurements taken in the studied population indicate high lean meat content in domestic fattening pigs reaching 58.94% according to the old regression equation and 59.52% according to the new equation (Table 10). The RMSEP errors for the CGM-Sydel device for the old and new regression equations are 2.16% and 2.22%. The carcasses are characterized by thin backfat and thick loin muscle. The highest carcass lean meat grades, i.e., E and S, were observed in 91.09% of the studied fattening pigs, and their share would be even higher if the structure (lean meat content) was determined on the basis of an equation from 2023 in comparison to an equation from 2011. The structure of the classes in the SEUROP system was changed in the pig population, which is well illustrated in the diagrams in Figure 4 and Figure 5.

## 4. Discussion

The material selected for the dissection testing of the CGM device was fully representative in terms of the national population of fattening pigs in mid-2023, as evidenced by the data presented in Table 1, Table 8, and Table 10. They show that the lean meat contents of both populations, i.e., domestic and selected for dissection, were 58.9% and 58.8%, the thicknesses of the backfat at the F1 point were 13.9 and 15.1 mm, and the hot carcass weights were 95 and 96 kg, respectively.

In the mass population, the majority of fatteners for slaughter in Poland are three- and four-breed crossbreeds and imported hybrid lines [12]. The import of piglets of these lines is constantly growing and now constitutes a significant part of the slaughter material in Poland. The import of live pigs in 2023 from January to November amounted to 6.8 million pigs [13]. The imported genetic material has a very high level of lean meat content, exceeding 60%, which means that the vast majority of pork carcasses from such exporting countries as Denmark, the Netherlands, and Germany are classified in the highest meat “S” class [6]. Already, in 2020, the share of S-class carcasses in the entire European Union was 62.5% [3].

This situation has a definite impact on the increase in the lean meat content in pork carcasses in Poland, which reached 59.0% in December 2023 [14], while at the time of the last testing of classification devices in 2011, it was lower at 55.4% [7]. In parallel with this progress, the average post-slaughter weight of carcasses also increased from 87.5 kg to 95.4 kg.

With the progress in terms of the slaughter value of fattening pigs, the testing procedure has changed. European Union experts have established that the simplified method of carcass dissection developed by Walstra and Merkus [4], although significantly correlated with the full dissection method, was not identical; thus, the results of both methods are not identical. That is why, in 2018, the obligation to develop a correction coefficient was introduced, called the scaling factor for the results of simplified dissection, which has to be developed on the basis of full and simplified dissection run in parallel [10].

This coefficient is determined each time when the equipment is tested based on the full and simplified dissection of part of the experimental group, and it is usually 10% of the experimental material, which is equal to 12 carcasses out of a total sample of 120.

In this study, the correction coefficient SF = 0.89 is obtained, which is the same as that recommended by the EU in 2008. The value of this coefficient depends primarily on the accuracy of the dissection. If its value is recommended by the EU, this means that it can be considered exemplary. It follows that the management team in this experiment performed the management work with high accuracy. This accuracy can vary up to approx. 2 pp between butchers performing dissection [3,15]. Olsen et al. [2] underlined, in their study, that the cutting of a hot carcass in half can be of great importance, especially in the calibration of new devices for classification and during dissection because the difference between the two halves of the carcass may reach 0.6 pp when cut wrongly. Since the SF coefficient value of 0.89 was achieved, it can be expected that the regression equations for CGM developed during the tests in 2011 and 2023 will give similar results for the lean meat content estimation of fattening pigs. Meanwhile, calculations performed on a large population of domestic fattening pigs taking into account both equations showed higher lean meat content using the 2023 equation, with the value increasing by an average of 0.58 pp. This shows that the form of the 2023 regression equation must have been influenced by factors other than just a change in the testing procedure including the determination of the SF coefficient. It has already been proven that the form of the regression equation, in addition to many other factors, is influenced by the level of lean meat content of fattening pigs, which increases with breeding progress [2,3,16,17,18].

In Poland, the lean meat content of fattening pigs increased by 3.6 pp in the studied period, and an increase in the post-slaughter weight of carcasses by 7.9 kg was also recorded (average hot carcass weight in 2011—87.5 kg [19]). In parallel with these features, the thickness of the back fat and LD muscle, i.e., the features included in the regression equation for the CGM device, have changed. This is why there is a need and justification to retest all of the remaining equipment operating in domestic slaughterhouses. The results of this research will be developed in a separate publication.

## 5. Conclusions

In summary, it should be stated that the new procedure for testing grading devices introduced by the EU in 2018 did not affect the results of dissection and, thus, the form of the regression equation for estimating lean meat content in Poland using the CGM-Sydel device, because when performing the dissection using the simplified W&M method, the correction coefficient SF = 0.89, used in EU regulations since 2008, was obtained. The equation was influenced by the increase in the level of lean meat content and fattening pig weight in the period from 2011 to 2023 in Poland. The implementation of the new equation will increase the lean meat content by 0.58 pp when using an optical-needle CGM device. The importance of the research is underlined by the fact that over 90% of pigs slaughtered in Poland have been classified in the SEUROP system.

## Figures and Tables

**Table 1:** The average weight of the carcass and backfat thickness at point F of the pig carcass population obtained in the 7 slaughterhouses (*n* = 882,475).

Traits	Average	SD
Hot carcass weight, kg	95.0	10.6
Backfat thickness in p. F, mm	13.9	4.3

**Table 2:** Carcasses divided into 3 groups based on the backfat thickness at point F.

Traits	Group 1	Group 2	Group 3
Backfat thickness, mm	0–10	11–18	>18
Number of carcasses	30	66	30

**Table 3:** Carcasses divided into 3 groups based on hot carcass weight.

Traits	Group A	Group B	Group C
Carcass weight, kg	60–84.4	84.5–105.5	>105.5
Number of carcasses	32	62	32

**Table 4:** The selection of the carcasses used in this study (in brackets with the required carcass numbers).

Dissection Type	Weight Carcass Groups	Backfat Thickness Groups			Total
		Group 1	Group 2	Group 3	
Partial dissection for total carcasses	A	7 (7)	18 (18)	6 (7)	31 (32)
	B	15 (16)	29 (30)	19 (16)	63 (62)
	C	6 (7)	17 (18)	9 (7)	32 (32)
	Total	28 (30)	64 (66)	34 (30)	126 (126)
Total dissection for part carcasses (subsamples)	A	1 (1)	2 (2)	1 (1)	4 (4)
	B	1 (1)	2 (2)	1 (1)	4 (4)
	C	1 (1)	2(2)	1 (1)	4 (4)
	Total	3 (3)	6 (6)	3 (3)	12 (12)

**Table 5:** Hot carcass weight and fat thickness (CGM) of selected materials in different fat subgroups.

Fat Groups	Carcass Weight, kg					Fat Thickness, mm				
	*n*	Mean	SD	Min.	Max.	*n*	Mean	SD	Min.	Max.
1	28	94.2	11.64	75.2	115.6	28	9.0	1.00	7.0	10.0
2	64	96.3	12.43	70.4	116.4	64	14.1	2.18	11.0	18.0
3	34	100.5	11.98	77.4	120.0	34	21.8	2.85	19.0	31.0

**Table 6:** Hot carcass weight and fat thickness (CGM) of selected materials in different weight subgroups.

Weight Groups	Carcass Weight, kg					Fat Thickness, mm				
	*n*	Mean	SD	Min.	Max.	*n*	Mean	SD	Min.	Max.
A	31	80.6	3.54	70.4	84.4	31	13.7	4.56	7.0	25.0
B	63	97.1	5.26	85.0	105.4	63	15.3	5.35	7.0	31.0
C	32	112.6	4.20	106.4	120.0	32	15.8	4.85	8.0	28.0

**Table 7:** Hot carcass weight and fat thickness (CGM) of selected materials in different gender groups.

Gender Groups	Carcass Weight, kg					Fat Thickness, mm				
	*n*	Mean	SD	Min.	Max.	*n*	Mean	SD	Min.	Max.
Gilts	63	96.3	13.7	70.4	120.0	63	15.1	5.1	7.0	28.0
Hogs	63	97.7	10.7	75.2	120.0	63	15.0	5.1	7.0	31.0

**Table 8:** Dissection results of referenced lean meat percentages in different dissection groups.

Dissection Groups	Lean Meat Content, %				
	*n*	Mean	SD	Min.	Max.
Fat group 1	28	63.1	2.25	55.8	66.8
Fat group 2	64	59.1	2.68	53.8	65.3
Fat group 3	34	54.6	3.06	48.7	61.8
Weight group A	31	58.3	4.23	48.8	66.8
Weight group B	63	58.7	4.10	48.7	65.9
Weight group C	32	59.3	3.67	51.8	65.5
Gilts	63	58.8	4.0	48.8	66.8
Hogs	63	58.7	4.0	48.7	65.9

**Table 9:** A comparison of the results of partial and total dissection carried out on the subgroup research population of carcasses (*n* = 12).

Dissection Method	Lean Meat Content, %				
	*n*	Mean	SD	Min.	Max.
Total	12	59.62	4.74	48.73	65.20
Partial without SF	12	67.12	4.98	54.84	72.57
Partial with SF	12	59.74	4.43	48.81	64.58

**Table 10:** The quality traits of the domestic pig population and meatiness stated by old (2011) and new (2023) regression equations.

Traits	Statistic Traits				
	*n*	Mean	Min.	Max.	SD
Hot carcass weight, kg	882,475	94.97	60.00	120.00	10.61
Loin thickness, mm	882,475	62.20	21.00	99.00	8.40
Fat thickness, mm	882,475	13.86	3.00	55.00	4.26
LMP, old equation, %	882,475	58.94	30.46	70.36	2.65
LMP, new equation, %	882,475	59.52	30.64	70.87	2.71

## Data Availability

The data of this study have been archived at the Department of Meat and Fat Technology, Prof. Wacław Dąbrowski Institute of Agricultural and Food Biotechnology—State Research Institute, Poznań, Poland.
